# Supplementary material for: Direct observation of coherence transfer and rotational-to-vibrational energy exchange in optically centrifuged CO2 super-rotors
Source: Nat Commun. 2023 Jun 3;14:3227. doi: 10.1038/s41467-023-38873-z (PMC10239519; doi:10.1038/s41467-023-38873-z)
Supplement: Supplementary file 1 — Supplementary Information [file 41467_2023_38873_MOESM1_ESM.pdf]

Supplementary Information for

Direct observation of coherence transfer and rotational-to-vibrational energy exchange in optically centrifuged CO_2

Timothy Y. Chen, Scott A. Steinmetz, Brian D. Patterson,
Ahren W. Jasper, Christopher J. Kliewer

Correspondence to: cjkliew@sandia.gov

This PDF file includes:

Supplementary Discussion
Figs. S1 to S10
Table S1

Supplementary Discussion

The adiabaticity parameter is calculated from the following expression:[1]

$$a = 2\pi\tau_c/T_J$$

where τ_c is the collision time determined by dividing the characteristic interaction length by the mean relative velocity and T_J is the molecular rotational period. The characteristic interaction length[2] with a value of 1.73 ± 0.04 Å. The adiabaticity parameter can be viewed as the mean angle through which a molecule will rotate during a collision within the characteristic interaction length.[3] Dividing by π gives the number of times a molecule rotates π rad, which due to the rotational symmetry of linear molecules like CO_2, we consider one rotation.

The main text references 5.5 eV as the bond dissociation energy limit for CO_2. This is the traditional thermal dissociation limit but is spin-forbidden. The singlet ground state of CO_2 has a much higher dissociation energy of 7.4 eV. Therefore, accessing the 5.5 eV limit requires a singlet-triplet interstate crossing transition as reported in the literature.[4] This is meant to qualitatively illustrate how high in the rotational energy ladder the centrifuged CO_2 has climbed (i.e. comparable to molecular bond energies). Therefore, we will not comment on whether this channel is open during the time scales of the experiment.

The criterion for determining the "onset time" for the vibrational hot bands was based on the earliest time a hot band peak was resolvable around $J = 35$. See Fig. S11 for examples. This is a qualitative measure intended to confirm the collisional nature of the hot band formation.

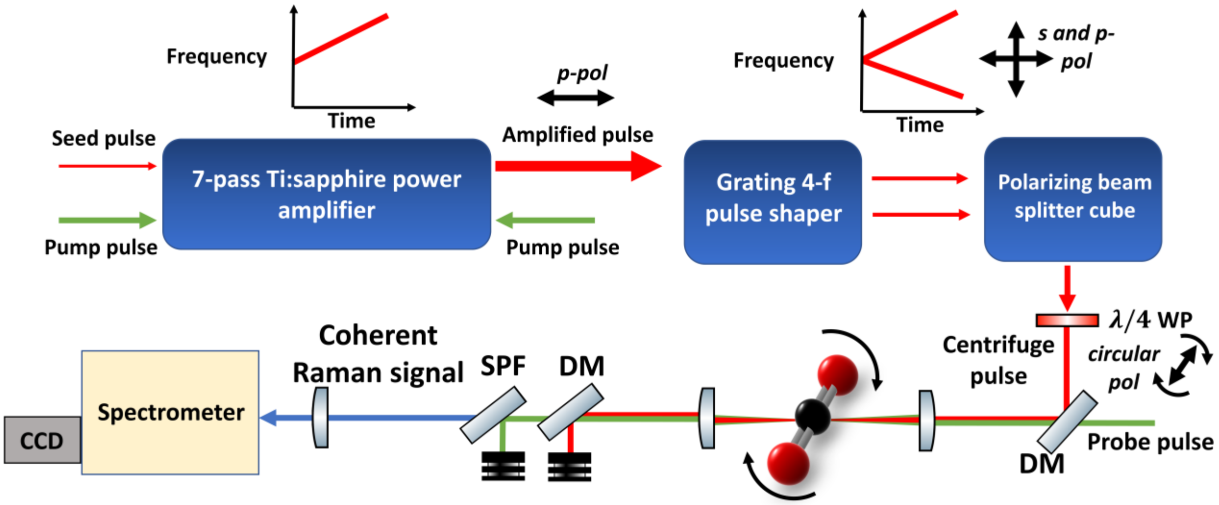

Fig. S1. Schematic of the optical centrifuge experimental setup.

The polarizations and frequency-time plots of the pulses at different stages of the apparatus are shown as reference. The following abbreviations were used. SPF: short pass filter, DM: dichroic mirror, WP: waveplate. A f = 100 mm achromatic lens was used to focus the centrifuge and probe pulses, while a f = 150 mm plano-convex was used to collimate the outgoing beams. The coherent Raman signal was focused into the spectrometer with a 75 mm focal length plano-convex lens.

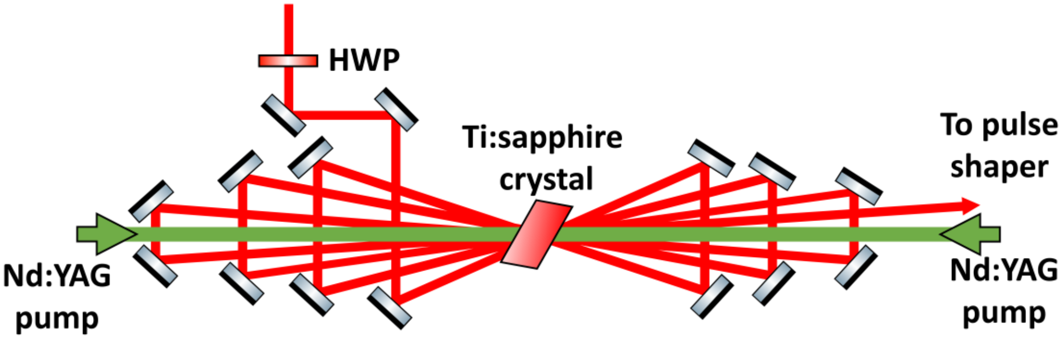

Fig. S2. Schematic of the 7-pass power amplifier.

The Ti:sapphire crystal housing was continuously cooled with 293 K water. HWP: half wave plate. End-pumping on both sides was pursued to prevent damage to the crystal.

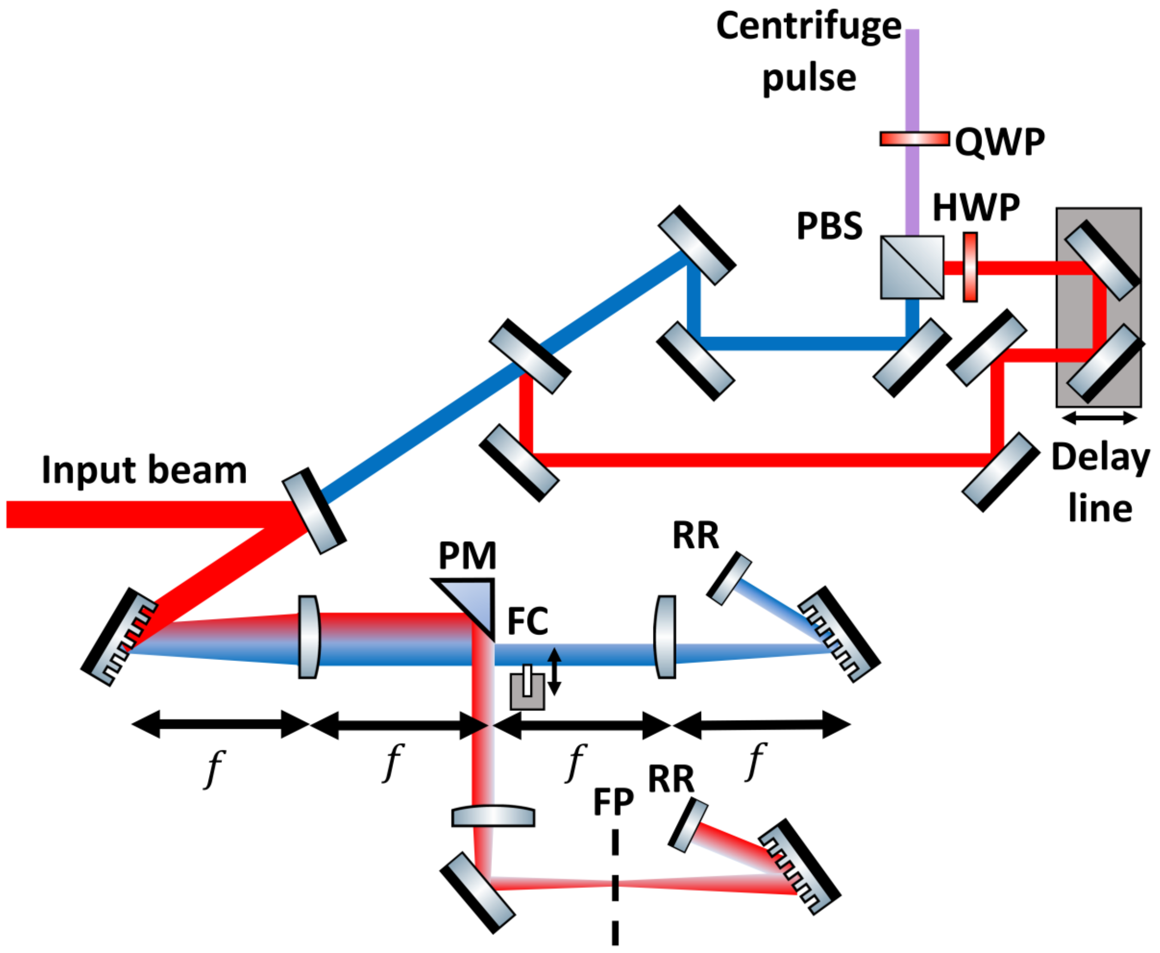

Fig. S3. Detailed schematic of the centrifuge pulse shaper.

PM: prism mirror, FC: frequency cutting stage, RR: retroreflector, FP: Fourier plane, PBS: polarizing beam splitter, HWP: half wave plate, QWP: quarter wave plate. The diffraction gratings were gold coated and had a line spacing of 1800 gr/mm. A motorized translation stage served as the delay line for fine temporal alignment of the two oppositely chirped arms.

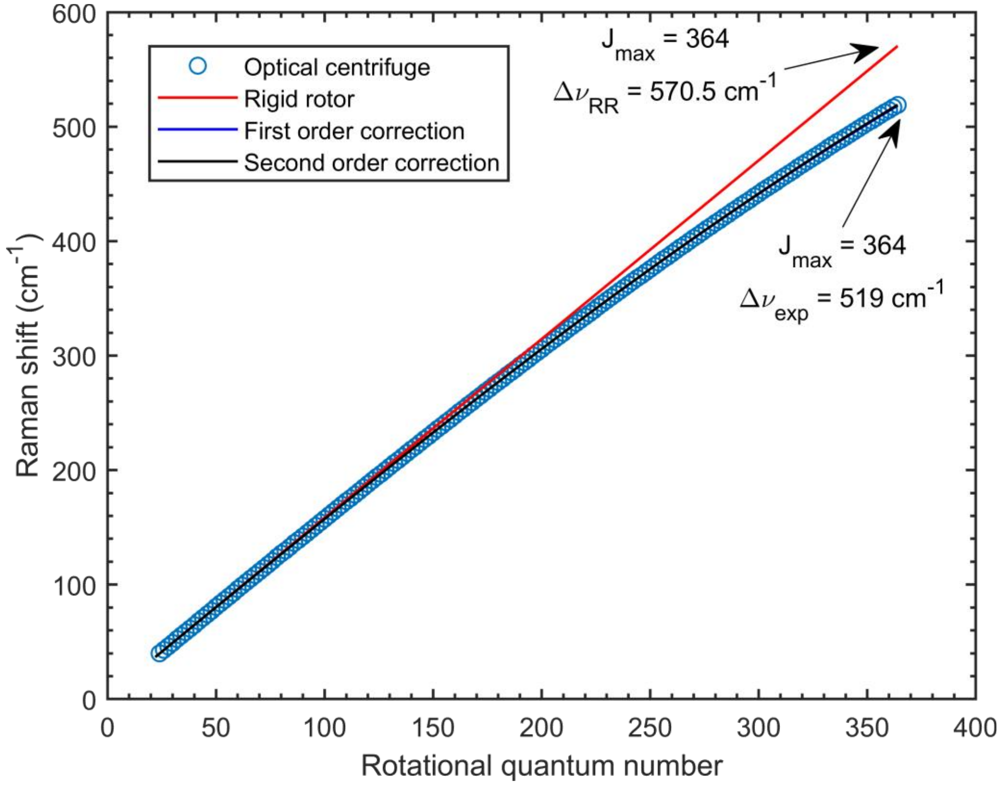

Fig. S4. Measured and predicted Raman shift.

Measured and predicted Raman shifts detected using an 1800 gr/mm grating spectrometer and different levels of centrifugal corrections. The difference between a rigid rotor calculation and the experiment reaches 61.5 cm^{-1}. The difference between the first and second order corrections cannot be seen on this scale.

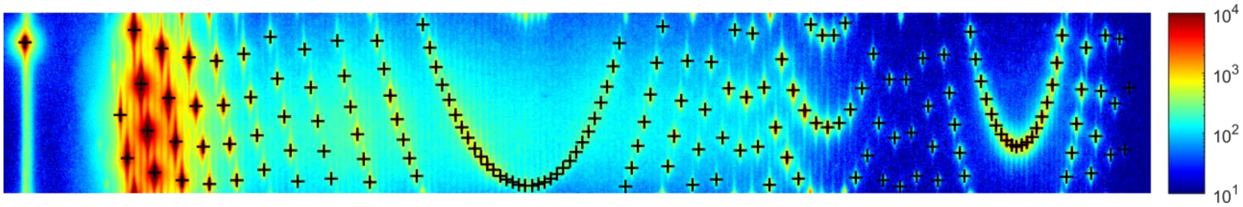

Fig. S5. VIPA spectrum of CO_2 super-rotors.

The measured 0.5 cm^{-1} FSR VIPA spectrum of CO_2 at 50 Torr. The extracted peak positions are marked with a cross. The limits in the vertical direction are approximately one free-spectral range apart.

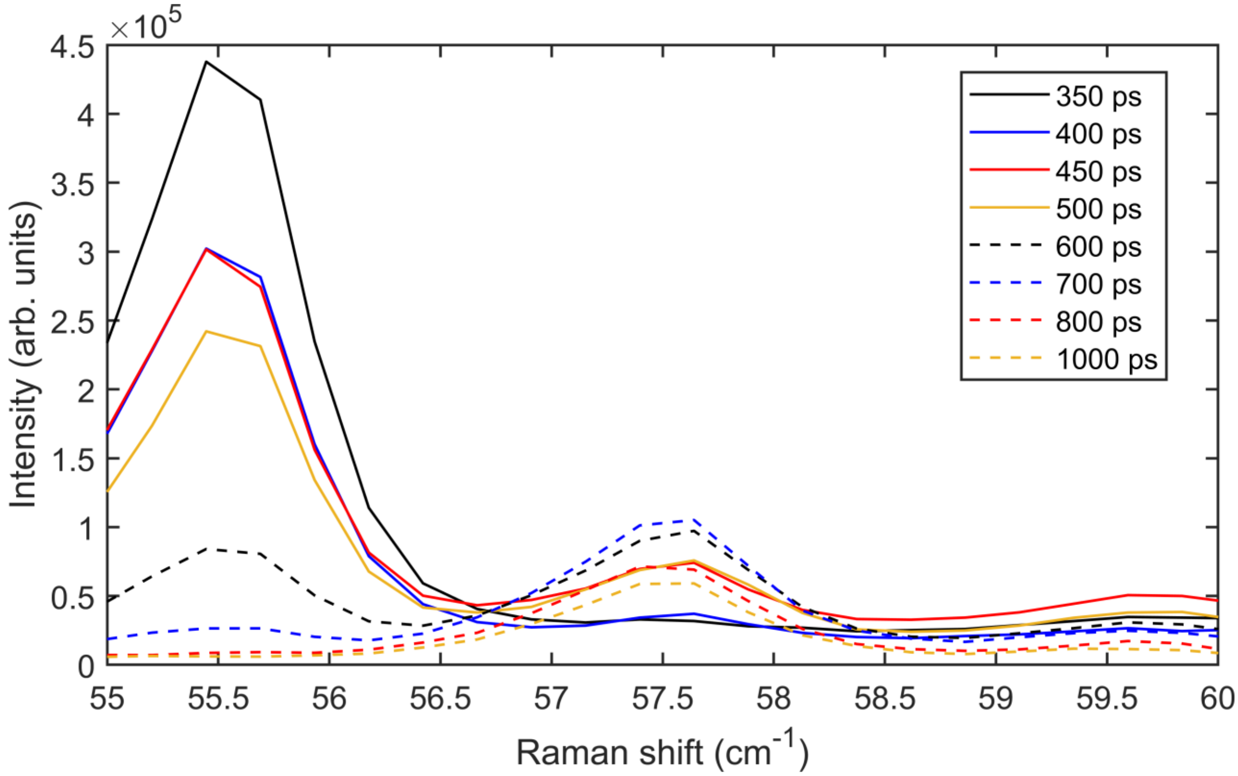

Fig. S6. Time-resolved VIPA spectra for CO_2 in a reduced bandwidth centrifuge.

A single row in the cut centrifuge VIPA images for the same conditions as in Fig. 3C and D centered on the J = 35 hot band transition for different picosecond probe delays. The hot band grows into the spectrum at a probe delay of 450 ps. The J = 34 ground state peak is visible on the lower frequency side.

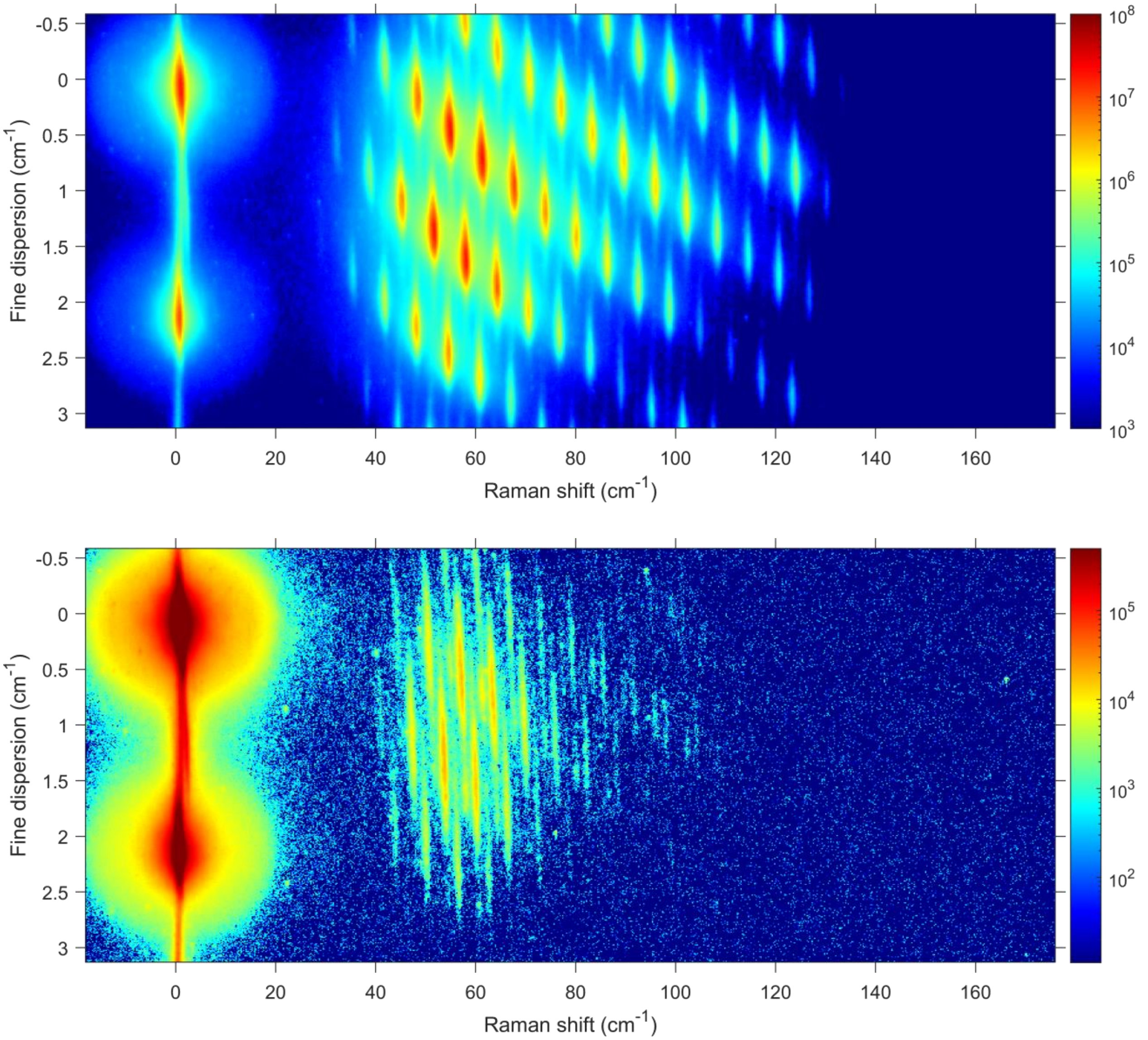

Fig. S7. Time-resolved VIPA images

VIPA images of centrifuged CO_2 with the centrifuge pulse terminated at a Raman shift of 126 cm^{-1} at 100 ps (top) and 700 ps (bottom) probe delays. Compared to Fig. 3D, the distribution of hot bands is narrowed and shifted to lower rotational levels.

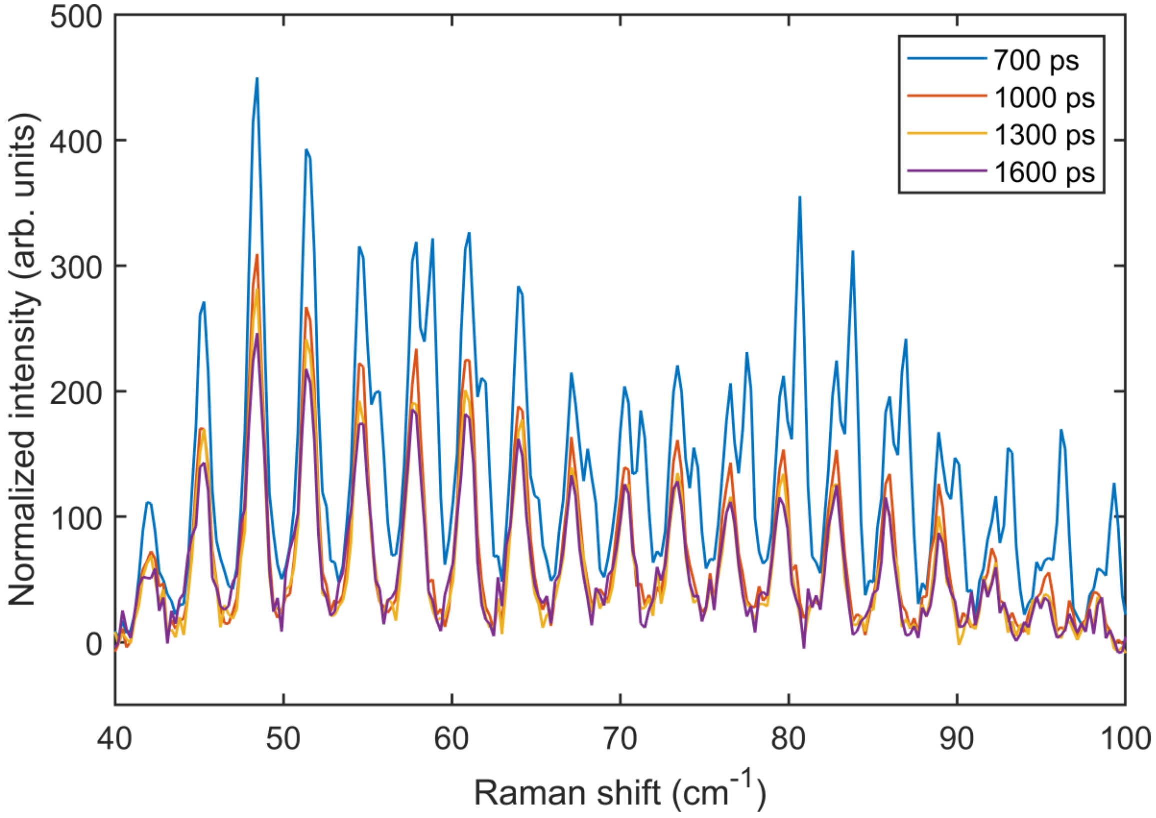

Fig. S8. Optically centrifuged CO_2 at 380 Torr probed from 700 ps to 1.6 ns.
Even at late probe delays, the hot bands are visible.

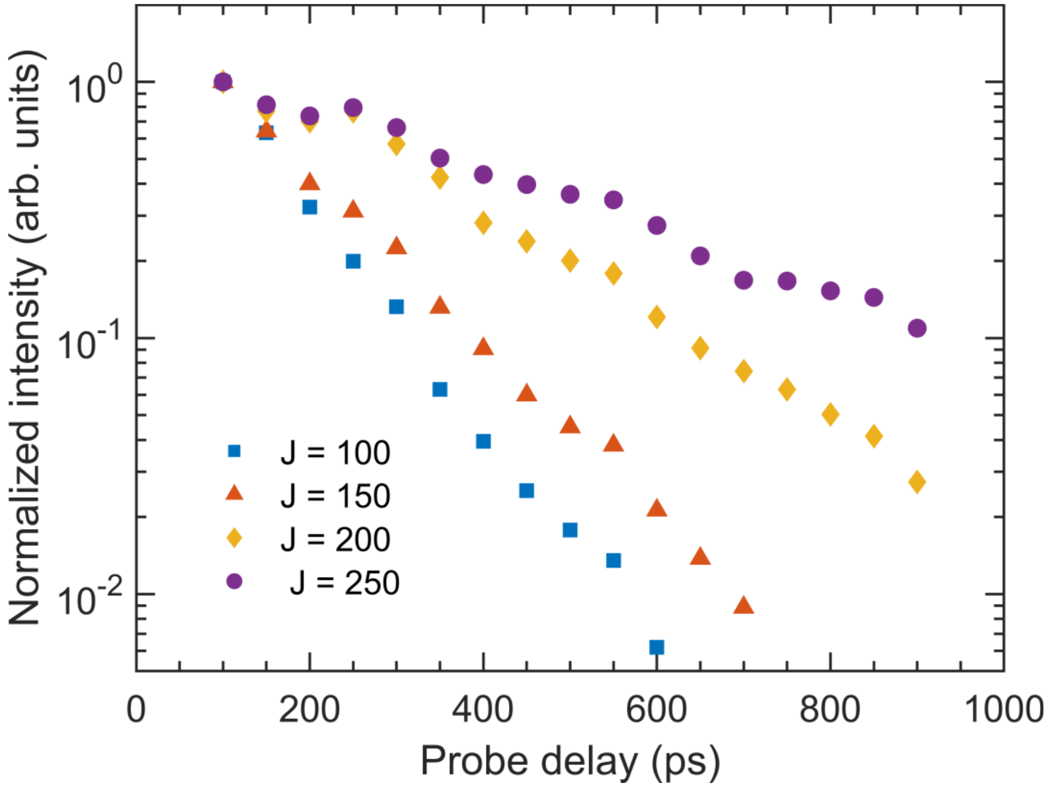

Fig. S9. Probe-delay scans indicate no explosive rotational-to-translational relaxation.
A single probe delay scan of optically centrifuged CO_2 at 380 Torr for different rotational levels plotted on a log scale. Two slopes indicative of explosive rotational-to-translational relaxation are not observed.

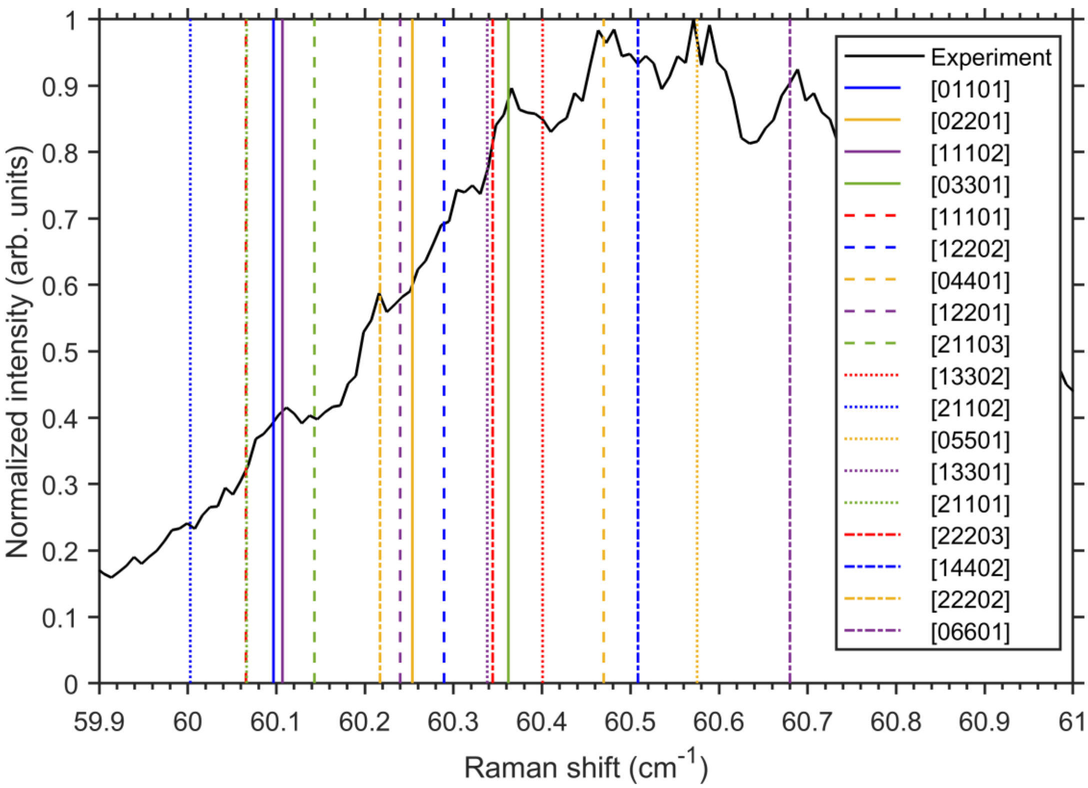

Fig. S10. Line-positions for vibrationally excited states of CO_2

Same as Fig. 4a, except all allowed rotational transitions from vibrationally excited states of CO_2 are shown. The legend follows the AFGL notation of $[\nu_1, \nu_2, l, \nu_3, r]$.

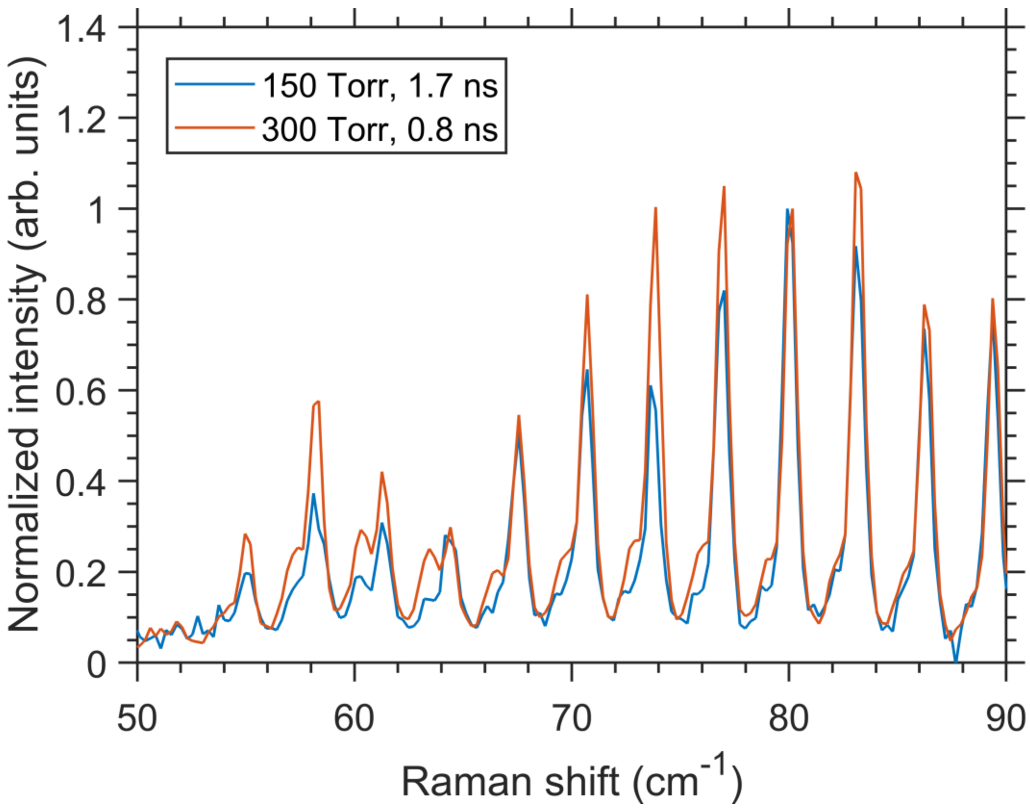

Fig. S11. Example spectra at 150 and 300 Torr used for determining the "onset time" of the vibrational hot bands.

The emergence of a distinguishable peak at low J was used as the criterion. The corresponding onset times were 1.7 and 0.8 ns for 150 and 300 Torr, respectively.

Table S1.

Fitted rotational and centrifugal distortion constants reported in the literature and determined in this study. Standard 1σ uncertainties are listed in parentheses in terms of the last reported digit. Note, the uncertainty in D determined here is two orders of magnitude higher than in other works while the uncertainty in L is one order of magnitude smaller.

	Rothman et al. 1992[5]	Long et al. 2013 [6]	Wu et al. 2020 [7]	Reed et al. 2021 [8]	Present work
B (cm^{-1})	0.39021889	0.390218955978 (648)	0.390218955978 (323)	0.390218957446 (244)	0.390218955978 (648)[1]
D (10^{-7} cm^{-1})	1.33338	1.33379886 (380)	1.33379919 (320)	1.33381214 (277)	1.333355 (215)
H (10^{-14} cm^{-1})	0.77	1.37305 (767)	1.3786 (117)	1.4203 (110)	1.3446 (316)
L (10^{-20} cm^{-1})	—	—	-1.87(140)	-6.27 (140)	-1.758 (126)

[1] The value from Long et al.[6] was used in this work.

Supplementary References

1 Milner, A. A., Korobenko, A., Hepburn, J. W. & Milner, V. Effects of Ultrafast Molecular Rotation on Collisional Decoherence. *Phys. Rev. Lett.* **113**, 5, doi:10.1103/PhysRevLett.113.043005 (2014).

2 Roy, S. *et al.* Direct measurements of collisionally broadened Raman linewidths of CO2 S-branch transitions. *J. Chem. Phys.* **138**, 6, doi:10.1063/1.4774093 (2013).

3 Korobenko, A., Milner, A. A. & Milner, V. Direct Observation, Study, and Control of Molecular Superrotors. *Phys. Rev. Lett.* **112**, 5, doi:10.1103/PhysRevLett.112.113004 (2014).

4 Hwang, D. Y. & Mebel, A. M. Ab initio study of spin-forbidden unimolecular decomposition of carbon dioxide. *Chem. Phys.* **256**, 169-176, doi:10.1016/s0301-0104(00)00108-7 (2000).

5 Rothman, L. S., Hawkins, R. L., Wattson, R. B. & Gamache, R. R. ENERGY-LEVELS, INTENSITIES, AND LINEWIDTHS OF ATMOSPHERIC CARBON-DIOXIDE BANDS. *J. Quant. Spectrosc. Radiat. Transf.* **48**, 537-566, doi:10.1016/0022-4073(92)90119-o (1992).

6 Long, D. A., Truong, G. W., Hodges, J. T. & Miller, C. E. Absolute (CO2)-C-12-O-16 transition frequencies at the kHz-level from 1.6 to 7.8 mu m. *J. Quant. Spectrosc. Radiat. Transf.* **130**, 112-115, doi:10.1016/j.jqsrt.2013.07.001 (2013).

7 Wu, H. *et al.* A well-isolated vibrational state of CO2 verified by near-infrared saturated spectroscopy with kHz accuracy. *Phys. Chem. Chem. Phys.* **22**, 2841-2848, doi:10.1039/c9cp05121j (2020).

8 Reed, Z. D., Drouin, B. J. & Hodges, J. T. Inclusion of the recoil shift in Doppler-broadened measurements of CO2 transition frequencies. *J. Quant. Spectrosc. Radiat. Transf.* **275**, 3, doi:10.1016/j.jqsrt.2021.107885 (2021).
